# From Clonal Hematopoiesis to Therapy-Related Myeloid Neoplasms: The Silent Way of Cancer Progression

**DOI:** 10.3390/biology10020128

**Published:** 2021-02-06

**Authors:** Carmelo Gurnari, Emiliano Fabiani, Giulia Falconi, Serena Travaglini, Tiziana Ottone, Antonio Cristiano, Maria Teresa Voso

**Affiliations:** 1 Department of Biomedicine and Prevention, University of Rome Tor Vergata, 00133 Rome, Italy; carmelogurnari31@gmail.com (C.G.); giulia_0312@hotmail.it (G.F.); serenatravaglini@live.it (S.T.); tizianaottone@hotmail.com (T.O.); cristianoantonio93@hotmail.it (A.C.); voso@med.uniroma2.it (M.T.V.); 2 Immunology, Molecular Medicine and Applied Biotechnology, University of Rome Tor Vergata, 00133 Rome, Italy; 3 Department of Translational Hematology and Oncology Research, Taussig Cancer Institute, Cleveland Clinic, Cleveland, OH 44195, USA; 4 Saint Camillus International, University of Health Sciences, 00131 Rome, Italy; 5 Laboratorio di Neuro-Oncoematologia, Fondazione Santa Lucia, 00179 Rome, Italy

**Keywords:** clonal hematopoiesis, therapy-related myeloid neoplasms, myeloid progression

## Abstract

**Simple Summary:**

In the last decades the improved management of cancer patients and the overall prolonged life expectancy contributed to the increased number of patients at risk of late clonal events such as therapy-related myeloid neoplasms (t-MN). The discovery of clonal hematopoiesis of indeterminate potential (CHIP) in normal individuals has shed light on the pathophysiologic mechanism behind the process of myeloid evolution, defining CHIP carriers at higher risk of progression. Moreover, different patterns of clonal evolution have been identified in case of t-MN development after anti-cancer treatment exposure. The growing body of evidence in this field allowed the creation of dedicated cancer survivorship programs and “CHIP-Clinics” in order to specifically address the issue of CHIP in patients undergoing anti-cancer treatment and develop measure of early detection possibly guiding tumor surveillance.

**Abstract:**

Clonal hematopoiesis (CH) has been recognized as a predisposing factor for the development of myeloid malignancies. Its detection has been reported at different frequencies across studies, based on the type of genome scanning approach used and the population studied, but the latest insights recognize its virtual ubiquitous presence in older individuals. The discovery of CH in recent years paved the way for a shift in the paradigm of our understanding of the biology of therapy-related myeloid malignancies (t-MNs). Indeed, we moved from the concept of a treatment-induced lesion to a model where CH precedes the commencement of any cancer-related treatment in patients who subsequently develop a t-MN. Invariant patterns of genes seem to contribute to the arising of t-MN cases, with differences regarding the type of treatment received. Here, we review the principal studies concerning CH, the relationship with myeloid progression and the mechanisms of secondary t-MN development.

## 1. Introduction 

The process of malignant transformation requires the stepwise acquisition of diverse genetic alterations over the course of many cell divisions [1,2]. Each tissue harbors premalignant populations of cells that possess only a subset of the lesions required for full-blown malignant transition, as in the case of atypical nevi or a colon polyp [3]. In adults, blood cell production is the result of the collective contribution of 5–20 × 10^4^ activated hematopoietic stem cells (HSCs) [4]. In normal physiological conditions, all stem cells contribute equally, while in the abnormal state of clonal hematopoiesis (CH), one or a few HSCs give rise to an imbalanced production of a large amount of cells at a disproportionate rate as compared to other clones [5]. Clonality generally indicates a population of related myeloid cells that can be identified by the presence of genetic alterations such as somatic mutations, copy number variations or cytogenetic aberrancies [6]. This phenomenon has a linear correlation with age and has been linked to higher odds of development of myeloid malignancies, with a risk of progression of 0.5–1% per year, cardiovascular events and all-causes mortality [7].

Besides age, other recognized risk factors for CH in healthy individuals are cigarette smoking, male sex and some genetic characteristics [7]. Indeed, CH rate seems to be lower in individual of Hispanic origin, also confirmed by the lower incidence of myelodysplastic syndromes (MDS) in this ethnicity, while germ-line polymorphisms of the *TERT* gene seem to be a risk factor [7,8]. 

Therapy-related myeloid malignancies (t-MNs) are an emerging problem of our aging societies, where newer therapies and ameliorated cancer management protocols are improving the life expectancy of patients diagnosed with either solid or hematologic cancers [9]. This means that an increasing number of patients treated with chemotherapy and/or radiotherapy will be at risk of developing this complication, characterized by abysmal prognosis and refractoriness to current treatment strategies and still remaining an unmet clinical need of cancer survivorship programs [10].

The hematopoietic tissue provides a fantastic lens into CH, and in recent years, several lines of evidence generated insightful clues as to the mechanism of myeloid neoplasms development from this early premalignant state. Here, we will focus on the principal studies on CH, its clinical manifestations, the mechanisms contributing to its development and the relationship with the occurrence of t-MN.

## 2. CHIP (Clonal Hematopoiesis of Indeterminate Potential) and ARCH (Age-Related Clonal Hematopoiesis): Discovery, Biology, Definition and Risk Factors

**Figure 1:**
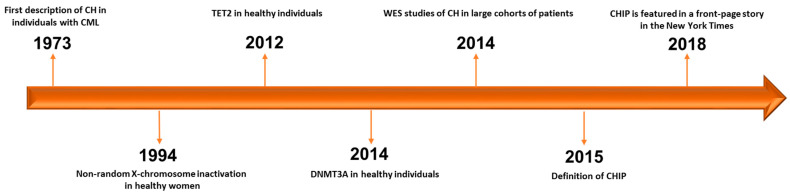
Timeline of progress towards discovery and definition of CHIP. CH: clonal hematopoiesis. CML: chronic myelogenous leukemia. WES: whole-exome sequencing. CHIP: clonal hematopoiesis of indeterminate potential [11,12,13,14,16,17,18,19].

Historically, the first example of CH dates back to 1973, when J. Rowley identified a chromosomal abnormality in nine patients with chronic myelogenous leukemia using quinacrine fluorescence and Giemsa staining [11] (Figure 1). Subsequently, in 1994, Fey et al. demonstrated the presence of CH associated to non-random X-chromosome inactivation in 21 of 105 healthy women [12]. Of note, CH resulted more frequent in elderly women (aged 75 to 96 years) than in younger women (aged 20 to 58 years) and healthy female children (aged 2 to 8 years) [12]. The first gene associated to this phenomenon in healthy individuals was *TET2*, followed by *DNMT3A* identified two years later [13,14]. In particular, mutations in *TET2* were found in about 5% (10 out of 182) of healthy elderly women with non-random X-chromosome inactivation by Busque et al. in 2012 [13]. It is noteworthy that *TET2* mutations are present in up to 25% of patients with a diagnosis of myeloid malignancy, and before 2012, no healthy individual had been found with mutations in recognized leukemia driver genes [13,15].

The improvement of genome scanning techniques gave rise, in 2014, to the discovery of a close association between HSC aging and the accumulation of somatic mutations in patients without hematological malignancies. Whole-exome sequencing (WES) studies on peripheral blood samples from large cohorts of non-hematological patients revealed a high incidence of mutations in three genes previously known to be frequently mutated in hematological malignancies [16,17,18]. These mutations were subsequently referred to as DTA, an acronym generated from the initials of the main genes involved (*DNMT3A*, *TET2* and *ASXL1*). Using data deriving from The Cancer Genome Atlas (TCGA) from 2700 blood samples used as germ-line controls across 11 different cancer types, Xie et al. found that mutations in *DNMT3A*, *TET2*, *ASXL1*, *JAK2*, *SF3B1*, *PPM1D* and *TP53* were enriched with a linear relationship with age [16]. However, the list of genes frequently mutated in the studied population did not encompass the full genomic landscape of myeloid leukemic driver genes, and mutations in *FLT3*, *NPM1* and *IDH1/2* were rarely found, suggesting a selective fitness advantage of lesions in some genes over others. Another two seminal studies confirmed these findings, further emphasizing the link between CH and age and linking this phenomenon to a higher risk of myeloid disorders, cardiovascular events, type 2 diabetes and overall mortality [18]. Of note, the incidence of *DNMT3A*, *TET2*, *ASXL1* and other mutations associated to HSC aging, such as *SF3B1*, *SRSF2*, *PRPF8*, *U2AF1*, *TP53*, *PPM1D*, *JAK2*, *GNAS*, *GNB1*, *CBL*, etc., was substantially rare in those under the age of 40 (<1%), but progressively higher in older individuals (about 20–30% of individuals aged 70 or older) [17,18]. Moreover, in these studies, the pattern of genes frequently mutated changed, with the exception of the DTA triad, always present at the highest frequency. In particular, the studies by Xie and Genovese et al. pointed out a previously not well-characterized gene called *PPM1D* (not included in the Jaiswal panel [18]), particularly enriched in individuals who received cancer treatments and subsequently developed a t-MN [16,17]. Following studies provided further evidence regarding the incidental and non-malignant significance of the persistence of *DNMT3A*, *TET2* and *ASXL1* mutations detectable by NGS after initial induction chemotherapy for acute myeloid leukemia (AML) [20,21,22]. 

As of today, it is well known that somatic mutations related to CH may affect only a small percentage of cells, resulting in a limited number of alleles carrying the somatic variant. Since the studies reported above used WES techniques, which are relatively insensitive to lower variant allele frequency (VAF) and, therefore, to smaller clones, newer genome scanning methods shed light on the real frequency of age-related mutations, which is greater than reported. Using targeted NGS panels at very high sensitivity, several authors have identified mutations at low VAF (<0.1%) in a high proportion of healthy people [23]. Since these mutations are present not only in patients with detectable hematologic disorders but also in healthy individuals, this phenomenon has been defined as age-related clonal hematopoiesis (ARCH), whereas clonal hematopoiesis of indeterminate potential (CHIP) is defined by the presence of somatic mutations with a VAF ≥2% [6,24]. 

Although the genes mutated in CH are the same ones commonly mutated in hematologic malignancies such as MDS, MDS/MPN (myeloproliferative neoplasms) and AML, the presence of these mutations alone, without other hematological manifestations, is not sufficient to fulfill the criteria for the diagnosis of a myeloid neoplasm. This notwithstanding, the aforementioned studies have reported an overall increased risk of transformation to hematological malignancy in patients carriers of CHIP-related mutations, with a risk of progression of about 0.5–1% per year vs. <0.1% in non-CHIP carriers [6,17,18]. Mechanistic and biological analogies with monoclonal gammopathy of undetermined significance (MGUS), a clinical scenario very well known by the hematology community, are obvious [25]. 

Different hypotheses have been proposed as to the mechanism of CHIP transitioning to overt myeloid neoplasia. Mutated CHIP genes are able to confer a proliferative or survival advantage to the affected cells, enabling a privileged clonal expansion, and the risk of progression in patients with CHIP has been found to be closely related to the clonal burden. Indeed, Jaiswal et al. reported that carriers of CHIP-related mutations with a VAF ≥10% had a higher risk of progression as compared to individuals with VAF <10% [18]. Besides VAF, the number of mutations positively correlates to the progression risk [3,26,27]. CHIP lesions may provide a survival advantage to clonal HSCs by retaining self-renewal capabilities and blocking differentiation (DTA triad gene mutations), and/or by potentiating the DNA damage response pathway without the activation of apoptosis (*TP53, PPM1D*) [5]. Cellular proliferation and/or survival advantages due to gene mutations may also explain the recent finding that CHIP-associated mutations have been identified in back-tracked samples from patients who developed an MDS after chemo/radio-therapy for a previous cancer or an autoimmune disease (t-MN) [28].

## 3. When Clonal Hematopoiesis Becomes Clinically Evident: The Cases of ICUS (Idiopathic Cytopenia of Undetermined Significance) and CCUS (Clonal Cytopenia of Undetermined Significance)

The shadowlands between CHIP and myeloid progression blur when patients present with clinical manifestations other than CHIP, such as cytopenias. Usually, this category of patients undergoes a wide and careful clinical laboratory evaluation, but in a fraction of these, still no final diagnosis of overt myeloid neoplasia can be made. Once other causes are ruled out, if the patient does not fulfill the WHO criteria for MDS, the most probable label is another four letter acronym: ICUS, idiopathic cytopenia of undetermined significance [29]. This acronym was suggested in 2007 for defining the diagnostic interface with lower-risk MDS [30].

However, what does define cytopenia? Patients with this diagnosis must have had, for at least 6 months, hemoglobin, platelet and neutrophil counts less than 11 g/dL, 100 × 10^9^/L and 1.5 × 10^9^/L, respectively [31]. These patients, after initial evaluation, need a careful “watch and wait” approach, by regularly monitoring complete blood counts (CBCs) due to the risk of myeloid progression. Indeed, it has been proven that already at the stage of ICUS, they may harbor CHIP [32]. When a patient with ICUS harbors a somatic mutation in myeloid genes but still does not fulfill the WHO-based MDS criteria, they are diagnosed with clonal cytopenia of undetermined significance, CCUS [33,34]. In the majority of cases, patients with persistent cytopenias undergo a bone marrow (BM) evaluation to rule out the presence of dysplasia, the morphological hallmark of MDS, or increased blast proportion. Many studies tried to propose objective criteria in order to eliminate, or at least minimize, inter-individual differences in evaluating BM smears [33]. Nevertheless, dysplasia features may also be detected in patients with disorders other than MDS, and microscopic evaluation is necessary but not sufficient for the definition of MDS diagnosis. Moreover, not only the assessment but also the quantification of dysplasia (the last WHO revision requires at least 10% of dysplastic cells in a given lineage) is extremely difficult and subjective [34,35]. Of note, dysplasia may also be found in patients with normal CBC. The situation of BM dysplasia in individuals without cytopenias, which may be co-occurrent with CHIP, defines another four letter acronym: IDUS, idiopathic dysplasia of undetermined significance (Table 1). 

As previously mentioned, about 30% of elderly people (aged 70 or above) harbor mutations in myeloid driver genes, with a risk of progression for CCUS much higher than for ICUS and CHIP [17,18]. Using a panel of 40 myeloid genes in a cohort of 683 patients with unexplained cytopenias, Malcovati et al. [36] demonstrated that 64% of patients carried a somatic mutation (CHIP) in at least one gene. Of note, patients with ICUS had a lower mutation rate (median number 0, range 0–7) when compared with patients with a WHO-diagnosed myeloid malignancy (median number 2, range 0–9, *p* < 0.001). The same trend was noticed when looking at VAF data, with the highest values in patients with myeloid neoplasms (median 34%, ranging from 2% to 100%) moving to lower burden of disease in patients with ICUS (median 27%, range 2–88%) and other non-ICUS cytopenias (median 5%, range 2–53%) (*p* < 0.001). The risk of progression was higher in patients harboring more mutations (especially in splicing machinery genes), VAF over 30% and in CCUS vs. ICUS patients (75% vs. 10% over a 5-year period, respectively) [36]. However, the progression rate also depends on the type of gene mutated. Many studies showed that mutations in the splicing genes *ASXL1*, *RUNX1* and *TP53* confer a higher progression rate and are associated with shorter latency before the overt MDS diagnosis [37]. Thus, the gap between CCUS patients and low risk MDS is extremely narrow, and the evaluation of cancer survivors who underwent chemo-/radiotherapy for a primary malignancy presenting with CHIP and cytopenias remains an anxiety-provoking scenario among hematologists. 

## 4. Therapy-Related Myeloid Neoplasm and Clonal Hematopoiesis: The Shift of a Paradigm

Therapy-related myeloid neoplasms (t-MNs) include AML and MDS arising in patients treated with chemo- and/or radiotherapy for a previous tumor or autoimmune disease. t-MNs represent one of the worst long-term consequences of anti-tumor treatments, as demonstrated by the poor survival outcomes (5-year overall survival <10%) [38,39]. In a recent analysis of data from the Surveillance Epidemiology and End Results (SEER) program, covering approximately 30% of the US patient population, Guru et al. [9] identified 1093 patients with a diagnosis of t-MN (median age of 65 years) among cancer survivors, resulting in an overall incidence of 0.13 cases/100,000 individuals, with variations based on age, race and period of diagnosis. The study showed that the incidence of t-MN increased in the last decade, probably as a result of better management of cancer patients. However, the overall survival (OS) of patients developing t-MN was extremely dismal, especially in older individuals, ranging from 51.3% at 2 years in the 20–39 age group to 19.3% in the 60–79 age group and 0% in patients aged >80 years [9].

Susceptibility factors for t-MN have been hypothesized for many years and included polymorphisms of detoxification and DNA damage repair enzymes, among others, but the role of these variants has never been confirmed by large studies, including appropriate controls [40,41]. Furthermore, germ-line variants of specific genes may contribute to the familiar recurrence of solid tumors, sometimes more than one neoplasm in the same individual, and t-MN [42,43,44].

CHIP mutations may represent a pre-malignant state in t-MN whose development can be triggered by exposure to cytotoxic damage. Two case–control studies demonstrated the role of pre-existing somatic mutations, prior to any chemotherapy, as a predisposing factor for t-MN [45,46]. Takahashi et al. [45], comparing 14 t-MN cases with 54 age-matched controls with lymphoma with a follow-up time of at least 5 years, detected CHIP in 71% of t-MN patients compared to 31% of controls (*p* = 0.008), with mutations in *RUNX1*, *TP53*, *SRSF2* and *TET2* genes more commonly observed in patients who developed t-MN. Similar results were obtained with an external validation cohort of patients with lymphoma treated with the CHOP regimen, with a 10-year cumulative incidence of t-MN of 29% in CHIP carriers vs. 0% in patients without CH (*p* = 0.009) [45].

A similar study focusing on elderly patients (≥70 years) was conducted by Gillis et al. [46] on 14 t-MN cases and 56 matched controls who had a history of previous chemotherapy exposure but did not develop t-MN. The authors demonstrated that cases of t-MN more likely harbored CHIP as compared to controls (62% vs. 27%, *p* = 0.024). The most commonly mutated genes in patients were *TET2* and *TP53* (each one accounting for 38%), while *TET2* (40%) was the most frequent in controls. In a cross sectional analysis of samples collected at the time of primary malignancy diagnosis and t-MN onset, the authors observed an expansion of VAF in the majority of carriers of CHIP lesions, while in one third, they registered a decrease, possibly suggesting that another mechanism of myeloid progression may be at play [46]. Moreover, when analyzing the VAF of CHIP lesions deriving from samples collected before or after chemotherapy start, no differences were seen, suggesting that these mutations were likely present before chemotherapy and eventually contributed to t-MN after exposure to cytotoxic stress [10]. As a matter of fact, this study focused specifically on elderly patients, who are characterized by an increased risk of carrying ARCH/CHIP lesions, possibly serving as a predictive biomarker for t-MN development in this specific setting.

In recent years, the results of these studies together with the data deriving from the frequency of CH in healthy individuals paved the way for an alternative t-MN pathogenesis. Indeed, cancer treatment may favor and select pre-existing CHIP lesions instead of directly being responsible for their development as we thought until last decade. However, as mentioned earlier, some CHIP mutations may actually decrease in VAF at the time of t-MN diagnosis, while others may remain unchanged or increase. For example, Arends and colleagues, studying clonal dynamics in 22 chemotherapy-treated patients, found that 40% of clonal mutations showed a change in VAF by 50% over time [47]. In particular, *DNMT3A* clones generally remained stable while clones harboring *RAD21*, *PPM1D* and *EZH2* mutations increased, and *SF3B1*, *JAK2* and *CBLB* clones decreased over time. In the same line, our group found that the majority (6/7) of patients with chronic lymphocytic leukemia (CLL) with detectable CHIP variants at t-MN diagnosis already possessed the same variants at the CLL phase with either lower (*n* = 4) or similar (*n* = 2) VAF [48]. Differences in patterns of genes involved in CHIP may be responsible for this phenomenon, with certain clones carrying one particular mutation presenting differential fitness advantages over time (a process similar to a Darwinian “war of clones”) based on the exposure to different environmental *noxae* represented by chemo- and/or radiotherapy [49].

In another study of 401 patients with non-Hodgkin’s lymphoma undergoing autologous stem cell transplant (AuSCT), CHIP was detected in 30% of cases prior to this procedure [50]. The 10-year cumulative incidence of t-MN development was 14.1% in patients with underlying CHIP vs. 4.3% in non-CHIP carriers (*p* = 0.0002). *TP53* and *PPM1D* were the most frequently mutated genes, and the risk was higher for patients carrying more than one CHIP lesion (25.3% at 10 years vs. 9.9% in patients with one lesion only, *p* < 0.001) [50].

Finally, the link between CHIP, cancer treatment and specific gene mutations was defined in a seminal study by Coombs et al. [51], where data of paired tumor and blood samples from 8810 individuals were used to dissect the role of CHIP in patients with solid tumors. Approximately 25% of patients were CHIP carriers at the time of cancer diagnosis and, in line with previous studies, this frequency was associated with cigarette smoke, linearly correlated with age and adversely influenced the overall survival of these patients affected, as said, by non-hematological malignancies [51]. Interestingly, *PPM1D* and *TP53* CHIP was associated with prior exposure to chemotherapy and increased risk for subsequent t-MN. Moreover, studying the mutational signatures of CHIP lesions, the authors highlighted that while C > T transitions, widely recognized as part of the aging process [52] and the principal mechanism of ARCH, were the most typical single-nucleotide substitutions among the coding mutations, C > A transitions were instead enriched in treatment-naïve smokers compared to treatment-naïve non-smokers, suggesting a smoke-imprinted molecular signature within the context of CHIP/ARCH [51].

The same mechanisms may underlie the onset of myeloid neoplasms due to environmental exposure. Observational data derived from atomic bomb survivors without a diagnosis of hematological malignancy reported that CH may be accelerated by radiation exposure. Indeed, Yoshida et al. observed peripheral blood monocytosis in atomic bomb survivors compared to non-exposed individuals. In particular, monocyte levels were found to be higher in survivors over 60 years of age and associated with increased all-cause mortality [53].

Altogether, these findings indicate that the presence of low-level clones with leukemia-driving mutations is a common age-related phenomenon. However, CHIP alone is insufficient to initiate clonal selection and expansion without the additional influence of other factors [54].

## 5. The Case of TP53 and PPM1D in Therapy-Related CHIP

As mentioned earlier, the use of cancer patient cohorts to study CHIP lesions highlighted the higher frequency of particular lesions such as *TP53* and *PPM1D* in these patients compared to healthy individuals. This prompted several investigators to explore specifically how cancer therapy may shape the pattern of mutations of CHIP genes, their fitness advantage and clonal dominance towards t-MN development.

Using data from the Memorial Sloan Kettering-Integrated Mutation Profiling of Actionable Cancer Targets (MSK-IMPACT) from 21,146 patients with different types of cancer, Bolton et al. [55] recently found that CHIP was present in 30% of patients at a median VAF of 5% (range 2–78%), with 31% of cases harboring more than one lesion. In line with previous data [16], the most frequently mutated genes were *DNMT3A*, *ASXL1* and *TET2* with an enrichment of variants in myeloid genes, which represented only 20% of the MSK-IMPACT panel. This finding highlights the fitness advantage in terms of improved self-renewal capabilities provided by these *bona fide* oncogenic mutations over other cancer-driver genes. The patients enrolled in this study previously exposed to any type of cancer therapy (cytotoxic, radiation, immunological or targeted therapies) had higher odds of harboring CHIP (odds ratio (OR) = 1.3, *p* = 1 × 10^−6^), similar to current and/or former smokers (OR = 1.1, *p* = 5 × 10^−3^) [55]. These two parameters, type of treatment and smoking, correlated with specific molecular subtypes of CHIP. In particular, mutations in DNA damage response (DDR) genes such as *TP53*, *PPM1D* and *CHEK2* were strongly associated with exposure to cancer treatment, while *ASXL1* lesions were typical of smokers. Furthermore, *PPM1D* mutations were associated with previous platinum derivatives, radionuclide, taxanes, topoisomerase II inhibitors and radiation exposure, whereas those of *TP53* were linked to platinum, radiation and taxanes, with DDR mutant clones outgrowing other clones at the time of t-MN onset. Conversely, in patients with non-DDR CHIP (i.e., *DNMT3A*), the clones outcompeted the DDR CHIP lesions if the patients was not subsequently exposed to any cancer treatment. Finally, Bolton et al. [55] concluded that among patients progressing to t-MN (40% harboring *TP53* mutations), 59% recapitulated at least one of the mutations present at the stage of CHIP, and in the majority of cases (91%), the transformation was preceded by acquisition of subsequent genetic lesions (*FLT3*, *RAS* genes pathway).

Therefore, there are at least two mechanisms by which *TP53* mutations occur in t-MN: (1) *TP53* clones are present in patients before the onset of chemotherapy as CHIP, and chemotherapy promotes clonal selection of pre-existing clones; (2) *TP53* mutant HSCs or the chemotherapy itself may directly induce DNA damage and leukemogenic *TP53* lesions [56]. The studies discussed above provide multiple evidence that *TP53* mutations are frequently present in patients prior to the administration of chemotherapy. Wong et al. [28] identified mutant *TP53* clones with a very low VAF (<0.001%) in four out of seven patients, 3–6 years before the development of t-MN. These clones are generally resistant to chemotherapy and, thus, had a selective advantage in the post-chemotherapy state [28,57,58]. In the same line, our group, in 2017, identified mutations at low VAF (<0.1%) not only in *TP53* but also in *ASXL1* [59]. Using a collection of follow-up samples from 14 patients with a primary hematologic malignancy who developed a secondary AML, our data showed that clonal evolution in t-MN is a heterogeneous process, with some somatic mutations (such as *TP53* and *ASXL1*) preceding cytotoxic treatment and possibly favoring leukemic development [59].

**Figure 2:**
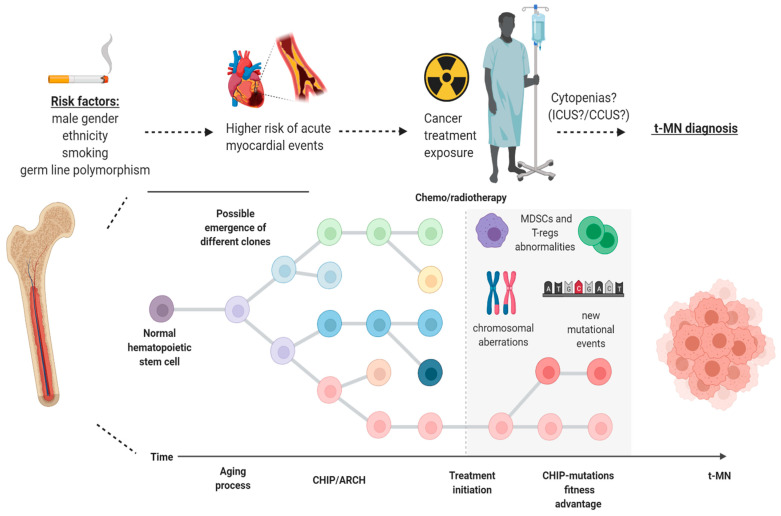
Model of progression of clonal hematopoiesis in patients with cancer undergoing treatment. Different risk factors (gender, cigarette smoking, ethnicity and germ-line mutations) may be at play, giving rise to clonal hematopoiesis of indeterminate potential (CHIP) in healthy individuals within the scenario of age-related clonal hematopoiesis (ARCH). After exposure to chemo-/radiotherapy, particular clones may have a fitness advantage over normal hematopoietic stem cells and, later on, through a stepwise process which may encompass the acquisition of new mutations, copy number alterations and MDSCs/T-regs abnormalities, can be responsible for the development of therapy-related myeloid neoplasms (t-MN). ICUS: idiopathic cytopenia of indeterminate potential; CCUS: clonal cytopenia of indeterminate potential; T-regs: regulatory T cells; MDSCs: myeloid-derived suppressor cells. Image was generated using BioRender.

Usually, *TP53*-mutated MNs are not characterized by single nucleotide variants only, but the mechanism of clonal progression to t-MN likely involves copy number alterations and the acquisition of other mutations [60]. Deeper sequencing of CHIP mutations within *TP53*-mutated samples demonstrated sub-clonal chromosome 5 and 7 copy number variations many years before the diagnosis of t-MN, suggesting that *TP53* clones precede the development of cytogenetic abnormalities in t-MN. Moreover, *TP53* clones expand over time and drive transformation to t-MN, being the bulk of the malignant clone at diagnosis [61]. This intrinsic advantage may be related to a specific *TP53*-related immune escape phenotype, as shown by a recent study where *TP53* mutant cases showed an imbalance of checkpoint molecules and expansion of highly immunosuppressive regulatory T cells and myeloid-derived suppressor cells [62] (Figure 2).

*PPM1D* is the second most frequently mutated gene, accounting for 20% of t-MN cases and, as mentioned, it has been linked to previous cancer treatment exposure [63]. In particular, DNA-damaging agents such as cisplatin are typically involved in selecting lesions in this gene [63]. *PPM1D*-mutated patients represent only <5% of *de novo* AML/MDS cases and, different from *TP53*, they usually do not present other detectable co-occurring chromosomal abnormalities. Mutations in this gene are usually truncating (either frameshift or nonsense), mapping within exon 6, with no particular hotspot. *PPM1D* truncating lesions generate an overexpression of the mutant protein, with a gain-of-function mechanism which constitutively inhibits DNA damage activation of p53 [63,64,65,66]. Hsu and colleagues [63] showed that *PPM1D*-mutated clones presented a growth advantage in case of cisplatin treatment, which was reversed after administration of GSK2830371, a PPM1D inhibitor, confirming the selective advantage provided by this mutation. As demonstrated by apoptosis assays, the authors also showed that this advantage derived by the acquisition of an “apoptosis-resistant” phenotype and that, once again, the administration of GSK2830371 was able to restore normal apoptotic levels in mutant cells. Finally, the specificity of platin-based chemotherapy over other types of cellular stress in selecting for *PPM1D* mutations was underlined by experimental data of competitive BM transplant in mice, in which *PPM1D* mutant cells showed reduced engraftment capacity, reconstituting the peripheral blood less effectively than wild-type counterparts [63].

## 6. Clinical Implications of CHIP: A Focus on Cardiovascular Risk

Since the first studies, the association of CHIP with cardiovascular events has been clear [18]. In particular, the risk of acute coronary syndromes is the same for CHIP carriers as for patients with uncontrolled dyslipidemia or smoking. In a large cohort of 4726 individuals with coronary heart disease and 3529 controls deriving from four case–control studies, Jaiswal et colleagues [8] showed that CHIP carriers had a hazard ratio (HR) for myocardial events 1.9 times higher than that of non-carriers (95% confidence interval (CI), 1.4 to 2.7) in two prospective cohorts, while the risk was even greater in the two retrospective cohorts (HR 4; 95% CI, 2.4–6.7). Of note, *DNMT3A*, *TET2*, *ASXL1* and *JAK2* mutations were associated with acute myocardial events which were linked to an increased coronary artery calcification process. This phenomenon has been related to the acquisition of endothelial dysfunction, as shown by mouse models of atherosclerosis lacking low-density lipoprotein (LDL) receptor. In these models, *TET2*-deficient macrophages showed upregulation of several inflammatory cytokines part of the NLRP3 inflammasome complex, which is known to contribute to both atherogenesis and MDS pathogenesis [67]. In particular, it has been shown that interleukin-1β upregulates the expression of P-selectin in endothelial cells, a known chemo-attracting agent for monocytes, currently evaluated as a possible actionable target in the setting of CHIP and MDS to decrease cardiovascular risk [68,69]. Another approach currently in evaluation is the use of vitamin C, taking into account the recent data on its role in restoring some TET2 functions [70,71]. However, as discussed, a growing body of evidence regarding the underlying link between CHIP and increased cardiovascular risk points towards the presence of deregulated inflammation, providing favorable conditions for the CHIP clone(s) to expand and produce hyper-inflammatory leukocytes/monocytes, ultimately leading to cardiovascular events [72]. The theory of the “Inflammaging”, a chronic exponential increase in pro-inflammatory cytokines with a consequential age-related decrease in stress response capability, may explain the emergence of CHIP lesions in the elderly (ARCH) [73]. This process, together with the deregulation of other immune cells such as regulatory T cells, myeloid-derived suppressor cells and natural killer (NK) cells, may perturb the homeostasis of the immune system, leading to a self-perpetuating cycle of CH and inflammation, which, for instance, characterizes the clinical phenotype of some myeloproliferative neoplasms [62,72]. Moreover, it may not be the case that knockout of one of the most commonly mutated CHIP and myeloid genes, *TET2*, leads to dysregulated development and proliferation of NK cells in mouse models [74].

As of today, some institutions that routinely use NGS panels to evaluate patients with both hematological and solid malignancies (with paired tumor and blood samples) have started to create dedicated “CHIP-Clinics” to establish the role of these mutations found in the blood compartment and guide clinical decisions regarding cancer treatment, myeloid progression surveillance and cardiovascular risk assessment [7]. However, it is noteworthy that no prospective trials specific for CHIP carriers have been performed so far regarding the measures of prevention and management of cardiovascular risk, and the current recommendations are based on those given by cardiologic societies for primary or secondary preventions of cardiac events in populations at risk.

## 7. Conclusions and Future Perspectives

Several challenges make the appropriate monitoring and prevention of t-MN in patients with CHIP a real conundrum of clinical practice. Patients with CHIP are usually retrospectively identified because of an antecedent diagnosis of malignancy (either solid or hematological). The presence of particular mutations may influence the choices for the treatment of the primary malignancy (i.e., the use of targeted therapies instead of platinum-based regimens in the case of *PPM1D* mutations, or the avoidance of AuSCT in patients with lymphoma and CHIP mutations). Moreover, a previous diagnosis of cancer has obvious psychological consequences for patients in otherwise complete remission from their primary tumors, but with a sword of Damocles represented by the risk of t-MN development because of the incidental finding of CHIP. It is not conceivable at the moment to routinely repeat multiple NGS tests over long time periods in all patients carrying CHIP because of the high costs and the psychological and economic burdens. However, this scenario highlights the importance of consensus-based guidelines and specific cancer survivorship programs, enabling the identification of high-risk CHIP carriers in need of more intensive monitoring and possibly taking advantage of intervention strategies in the context of clinical studies.

## Figures and Tables

**Table 1:** Acronyms describing various scenarios of clonal hematopoiesis.

Acronym	Condition	Description/Definition
ARCH	Age-related clonal hematopoiesis	Defined by detectable clonal hematopoiesis (marked by the presence of somatic mutations in the blood or bone marrow) occurring in elderly individuals.A specific VAF cut-off has not been defined and the clinical significance is undefined.
CHIP	Clonal hematopoiesis of indeterminate potential	Defined by somatic mutations of driver myeloid genes in the blood or bone marrow, present at ≥2% VAF in individuals without a WHO-defined hematologic disorder.
IDUS	Idiopathic dysplasia of undetermined significance	The finding of unexplained morphologic dysplasia of blood cells in individuals who are not cytopenic (also within clonal hematopoiesis).
ICUS	Idiopathic cytopenia of undetermined significance	Unexplained cytopenia(s) in patients who do not meet the diagnostic criteria for a myelodysplastic syndrome or other WHO-defined hematologic disorders (also within clonal hematopoiesis).
CCUS	Clonal cytopenia of undetermined significance	Unexplained cytopenia(s) in patients who do not meet the diagnostic criteria for a myelodysplastic syndrome or other WHO-defined hematologic disorders, but have somatic mutations of driver myeloid genes in the blood or bone marrow, present at ≥2% VAF.

## List of Gene Symbol Abbreviations

Gene symbol and official names follow the nomenclature of the GeneCards [75].

**Gene Symbol**	**Gene Name**
*ASXL1*	Additional sex combs-like 1
*CBL*	Casitas B-lineage Lymphoma Proto-Oncogene
*CBLB*	Cbl Proto-Oncogene B
*CHEK2*	Checkpoint kinase 2
*DNMT3A*	DNA Methyltransferase 3 Alpha
*EZH2*	Enhancer Of Zeste 2 Polycomb Repressive Complex 2 Subunit
*FLT3*	Fms-like tyrosine kinase 3
*GNAS*	Guanine nucleotide-binding protein G(s) subunit alpha
*GNB1*	Guanine nucleotide-binding protein G(I)/G(S)/G(T) subunit beta-1
*IDH1/2*	Isocitrate dehydrogenase 1/2
*JAK2*	Janus kinase 2
*NPM1*	Nucleophosmin
*PPM1D*	Protein Phosphatase, Mg^2+^/Mn^2+^ Dependent 1D
*PRPF8*	Pre-MRNA Processing Factor 8
*RAD21*	RAD21 Cohesin Complex Component
*RAS*	Rat Sarcoma Viral Oncogene Homolog
*RUNX1*	Runt-related transcription factor 1
*SF3B1*	Splicing factor 3B subunit 1
*SRSF2*	Serine And Arginine Rich Splicing Factor 2
*TERT*	Telomerase Reverse Transcriptase
*TET2*	Tet methylcytosine dioxygenase 2
*TP53*	Tumor protein p53
*U2AF1*	U2 Small Nuclear RNA Auxiliary Factor 1
